# Oral Health and Idiopathic Female Infertility: A Potential Association

**DOI:** 10.3290/j.ohpd.c_2670

**Published:** 2026-05-20

**Authors:** Dóra Kovács, Krisztina Boda, Attila Badó, István Gorzó, Norbert Pásztor, Márta Radnai

**Affiliations:** a Dóra Kovács Assistant Lecturer, Department of Prosthodontics, Faculty of Dentistry, University of Szeged, Szeged, Hungary. Data collection, dental examination, literature review, data interpretation, and manuscript writing.; b Krisztina Boda Honorary Professor, Department of Medical Physics and Informatics, Medical Faculty, University of Szeged, Szeged, Hungary. Statistical analysis, data interpretation.; c Attila Badó Gynaecologist, Obstetrics and Gynaecology, Medical Faculty, University of Szeged, Szeged, Hungary. Gynaecological examination, selection of patients, and manuscript revision.; d István Gorzó Professor Emeritus, Department of Periodontology, Faculty of Dentistry, University of Szeged, Szeged, Hungary. Supervision, manuscript revision and editing.; e Norbert Pásztor University Associate Professor, Obstetrics and Gynaecology, Medical Faculty, University of Szeged, Szeged, Hungary. Conceptualisation, idea of the study, study design, methodology, and manuscript revision.; f Márta Radnai University Professor, Department of Prosthodontics, Faculty of Dentistry, University of Szeged, Szeged, Hungary. Study design, conceptualisation, data interpretation, manuscript revision and editing.

**Keywords:** caries status, female infertility, oral health, periodontal status, periodontitis

## Abstract

**Purpose:**

To explore the possible link between periodontal and dental health and idiopathic female infertility in South East Hungary, along with identifying relevant sociodemographic factors.

**Methods and Materials:**

The study included women with idiopathic infertility and pregnant women (controls). All participants underwent a dental and periodontal examination, alongside a gynaecological assessment. Data were analysed by group (infertile vs control) and by age, education, and residence.

**Results:**

The study included 85 infertile and 65 pregnant women. Infertile women were older (34.5 vs 31 years, *P* < 0.001). Plaque scores were higher in controls compared to the infertile group (0.62 vs 0.52, *P* = 0.028), but there were no significant differences in bleeding on probing (BOP), probing depth, or body mass index. No group differences were observed for BOP ≥ 50% or ≥ 4 mm pocket depth. Significant differences were found in the number of filled teeth (*P* = 0.017) and decayed, missing, filled surfaces (DMFS) (*P* = 0.022). Educational level and residence impacted periodontal outcomes, with lower education and rural residence associated with poorer results.

**Conclusion:**

This study found no significant clinical differences between infertile women and pregnant controls regarding periodontal disease. While sociodemographic variations were noted, the results highlight the importance of dental care during pregnancy. Further research is needed to confirm these findings.

Infertility and periodontitis are two relevant health conditions affecting individuals worldwide. Recent research has suggested a potential connection between these seemingly unrelated conditions. This study was undertaken to evaluate the potential association between periodontal, dental status and idiopathic female infertility in a group of women in South East Hungary, as well as to identify sociodemographical factors that may contribute to infertility. Considering that periodontal disease serves as a risk factor for numerous health conditions, we hypothesised a link between infertility and periodontal disease. Our hypothesis suggested that periodontal disease negatively influences conception.

## Background

The World Health Organization (WHO) conducted multicenter studies to identify infertility aetiologies based on gender. It was shown that in 37% of the infertile couples, female infertility was the reason, in 8% of couples, male factor infertility, while in 35% of infertile couples, both male and female infertility were present.^36^

## Definition, Prevalence, and Evaluation of Infertility

Infertility refers to a state characterised by the inability to achieve a clinical pregnancy after engaging in regular, unprotected sexual intercourse for a period of 12 months or due to an individual’s or a couple’s inability to reproduce.^42^ Infertility imposes a significant emotional strain on couples desiring to conceive a child.^24^ The prevalence of infertility is about 13% among women and 10 % among men.^5^ It is affecting globally about 48.5 million couples.^22^ Recent studies have shown that infertile women are prone to other associated health conditions, such as depression and anxiety. According to a study, about 41% of infertile women suffered from depression and 87% from anxiety in the study group.^29^

## Periodontitis

Various inflammatory conditions affecting the supporting tissues around the teeth are collectively known as periodontal disease.^7^

Periodontitis is a condition characterised by inflammation caused by a combination of microbes and the body’s response to them. This inflammation causes attachment loss and loss of periodontal structures. The disease’s underlying processes involve specific molecular pathways. Ultimately, this leads to the production of proteinases produced by the host, which contribute to the breakdown of fibres in the periodontal ligament. Consequently, the junctional epithelium moves towards the root, allowing bacterial biofilm to spread down along the root surface.^34^

## The Impact of Periodontitis on General Health

Periodontitis is linked to multiple systemic conditions, such as negative outcomes during pregnancy,^12^ preterm birth,^28^ type 2 diabetes mellitus,^27^ increased morbidity of lower airway infections, notably pneumonia in individuals,^6^ cardiovascular diseases,^32^ respiratory illnesses,^31^ chronic kidney disease, and metabolic syndrome. Moreover, severe chronic periodontitis might be connected to initial atherosclerosis stages due to potential endothelial and microvascular dysfunctions.^18^

Some research has confirmed that certain indicators of chronic periodontal inflammation, including gingival bleeding, BOP, plaque and calculus accumulation, might be linked to oligo- and asthenozoospermia in men dealing with idiopathic infertility.^13,26^

Until now, a limited number of *in vivo* studies have investigated the potential connection between oral health status and female infertility, with a scarcity of published studies on this specific aspect.

### METHODS AND MATERIALS

#### Study Design

This prospective, non-randomised observational study took place at the Department of Obstetrics and Gynaecology, University of Szeged, Hungary, in coordination with the Department of Prosthodontics, University of Szeged, Hungary.

#### Study Population

Women of reproductive age with infertility were recruited and examined between March 2018 and September 2020, at the Department of Obstetrics and Gynaecology, University of Szeged, Hungary. Infertility was defined as a failure to achieve pregnancy despite unprotected, regular sexual intercourse during a period of 1 year. The fertility evaluation of the study group was conducted in line with the recommendations of the Minister of State for Health and the Hungarian Ministry of Human Resources. The recommendations were collected and published in a Hungarian National Guideline on the Investigation of Infertility and Subfertility and on Assisted Reproductive Techniques.^8^ The fertility evaluation contained medical history, gynaecological physical examination, transvaginal ultrasound examination, and antral follicle counting. Hormonal measurements were performed, including follicle-stimulating hormone (FSH), luteinising hormone (LH), thyroid-stimulating hormone, prolactin, estradiol, sexual hormone binding globuline, testosterone, dehydro-epiandrosterone, and 16-hydroxyprogesterone in the early follicular phase (3rd to 5th day of menstrual cycle). In case of a low number of antral follicles (< 5), anti-Müllerian hormone (AMH) measurement was performed. Glucose metabolism was assessed with an oral glucose tolerance test (at 0, 60, 120 min) with measurement of fasting insulin level. The shape of the uterine cavity and the patency of the Fallopian tubes were evaluated with hysterosalpingography. Male factor was assessed with sperm analysis according to the recommendations for the examination of human semen, released in 2010 by the World Health Organization.^37^

After the fertility evaluation of the couple, only patients with idiopathic infertility were enrolled in this study. According to the results of the fertility evaluation, exclusion criteria were: (1), untreated hormonal disorder (eg, polycystic ovary syndrome, hyperprolactinaemia, thyroid gland dysfunction, corpus luteum insufficiency, etc.); (2), low ovarian reserve (elevated FSH level according to the local laboratory, or AMH level below 1.2 ng/mL^25^; (3) any untreated disorder of glucose metabolism (defined as: insulin resistance: HOMA index ≥ 4^23^; impaired fasting glucose: 6.1–6.9 mmol/L; impaired glucose tolerance: glucose 7.8–11.0 mmol/L at 120 min; diabetes mellitus: fasting glucose ≥ 7.0 mmol/L, or any glucose level ≥ 11.1 mmol/L^39^; (4) abnormal shape of uterine cavity; (5) blocked or missing Fallopian tubes on both sides; (6) ultrasound signs of endometriosis; (7) abnormal semen results indicating male infertility. Further exlusion criteria were: (8) age below 18 or over 40; (9) previous periodontal therapy within 6 months; (10) antibiotic or NSAID therapy in the last month or at the time of the examination; (11) significant chronic systemic conditions (eg, autoimmune diseases, inflammatory bowel diseases etc.); (12) patients who need antibiotic prophylaxis for periodontal examinations; (13) refusal for participation.

Women in the first trimester of their complication-free pregnancy (week 11–12), supported by ultrasound examination results, and presenting in the Department of Obstetrics and Gynaecology to receive maternity care were included in the control group. Pregnant women after assisted reproductive treatments were not included. Each patient in the control group received all information regarding the dental examination, and their informed consent was obtained. Since the Hungarian antenatal care protocol mandates a dental examination during pregnancy, participation in our dental assessment was accepted in the majority of cases.

#### Information on Sociodemographic Data and Lifestyle Factors

Participants completed a questionnaire on lifestyle factors, including harmful habits such as smoking and alcohol consumption (during or before pregnancy). BMI was calculated based on the participants’ weight and height. Education levels were divided into four groups: primary school, technical school, high school, and higher education (university). Profession was categorised as unemployed, manual worker, intellectual worker, or other. Residence was classified as village or city.

#### Dental Examination

The dental examinations adhered to the guidelines established by the WHO in 1987.^38^ The recorded parameters included information on caries, radices, missing teeth, prosthetic restorations and fillings. Indices were calculated, such as decayed, missing, filled teeth (DMFT) and DMFS, with retained roots and teeth restored with crowns, considered as carious and/or filled surfaces. The dental examination was performed under clinical conditions in a dental surgery.

#### Periodontal Examination

A thorough periodontal examination, including probing and charting were carried out. The plaque was recorded (Silness–Löe plaque index), as well as the presence or absence of dental calculus, regarding each tooth. Bleeding on probing was considered positive if bleeding was observed within 15 s after probing at any site of the tooth. This occurrence was recorded in a dichotomous manner, indicating either the presence or absence of bleeding.

Probing depth was determined by measuring the distance from the gingival margin to the most apical portion of the gingival sulcus, considering six sites of each tooth (distobuccal, midbuccal, mesiobuccal, mesiolingual, midlingual, distolingual). This assessment was conducted using a millimetre-scale Michigan periodontal probe (Hu-Friedy, USA). Measurements were recorded in millimetres and rounded down to the nearest whole millimetre.

Wisdom teeth and radices were excluded from the periodontal charting in cases where it was not feasible to conduct probing depth measurements.

Poor periodontal status was defined by meeting two criteria: having a probing depth (PD) of ≥ 4 mm at least at one site of the teeth and the presence of BOP ≥ 50% of teeth.^17^

In the study, an experienced dentist carried out the periodontal screening, measuring the various parameters three times for each patient, aiming to eliminate inter-observer errors. The examiner also assessed intra-observer errors by taking repeated measurements of certain periodontal parameters at the start of the study, spaced 60 min apart. The results, expressed as intra-class correlation coefficients, demonstrated excellent agreement (> 0.90) for all examined periodontal parameters. This underscores the high reliability and consistency of the measurements throughout the study.

The current study was approved by the Regional and Institutional Human Medical Biological Research Ethics Committee in Szeged (Protocol number: 16/2018-SZTE). Each participant received all information regarding the examination, and their informed consent was obtained prior to the examination.

#### Statistical Analyses

Categorical variables are characterised by frequencies and percentages, and continuous variables are summarised by mean and standard deviation. Frequency distribution of categorical variables in control and infertile groups was compared by the Chi-square test, while averages of the continuous variables were compared by Student’s t-test. A comparison of dental and periodontal parameters in several groups (age groups, educational levels, profession, residence) was analysed by one-way ANOVA. To examine the potential risk factors of infertility, a multiple binary logistic regression was applied with infertility (yes or no) as the dependent variable and with sociodemographic, periodontal and dental data as the independent variables. The final model was gained by backward elimination, and the results are given as *P* values and adjusted odds ratios with 95% confidence intervals. SPSS version 29 was used for calculations. *P* values < 0.05 were considered to be statistically significant.

### RESULTS

In total, our study involved a cohort of 150 women, comprising 85 individuals experiencing infertility and 65 pregnant women serving as the control group. While our initial examination encompassed a broader spectrum of participants, the stringent application of exclusion criteria necessitated the omission of several individuals from our analysis.

In analysing the sociodemographic data, significant differences were discovered, particularly in age and alcohol consumption. The average age of infertile women was 34.5 years, whereas the control group averaged 31 years, indicating a statistically significant gap (*P* < 0.001). 56.5 % of infertile women admitted to consuming alcohol (occasionally), whereas in the case of pregnant women, this number is 24.6 %, which represents a significant difference. However, no significant difference was detected regarding place of residence (*P *= 0.247), BMI (*P* = 0.49), educational level (*P* = 0.974), or smoking habits (*P* = 0.18) between the two groups (Table 1).

**Table 1:** Sociodemographic and lifestyle data gained from the self-reported questionnaire

Sociodemographic and lifestyle factors	Infertile n = 85	Control n = 65	*P* value
**Age (average, yrs)**	34.50 ± 4.95 (min: 18–max: 40)	31.05 ± 5.59 (min: 18–max: 40)	**< 0.001**
**Place of residence**			0.247
City	62 (72.9%)	53 (81.5%)	
Village	23 (27.1%)	12 (18.5%)	
**BMI**	25.7 ± 6.27	25 ± 6.00	0.49
**Educational level**			0.974
Primary school	3 (3.5%)	2 (3.1%)	
Technical school	11 (12.9%)	9 (13.8%)	
Grammar School	25 (29.4%)	21 (32.3%)	
Higher education	46 (54.1%)	33 (50.8%)	
**Smoking**			0.180
Yes	12 (14.1%)	4 (6.2%)	
No	73 (85.9%)	61 (93.8%)	
**Alcohol consumption**			**< 0.001**
Yes, sometimes	48 (56.5%)	16 (24.6%)	
No	37 (43.5%)	49 (75.4%)	


**Fig 1:**
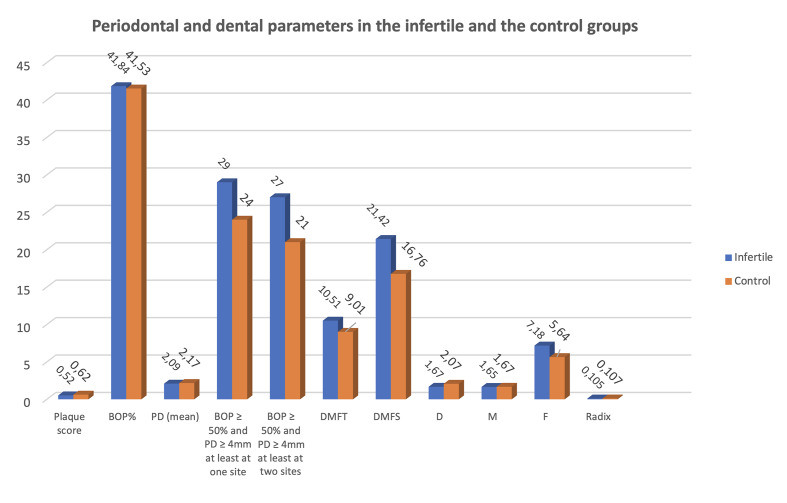
Results of the periodontal and dental examination in the infertile and control groups. PD: probing depth, BOP%: represents the percentage of teeth exhibiting bleeding on probing relative to those without bleeding. DMFT: decayed, missing, filled teeth, D: average number of decayed teeth, M: average number of missing teeth, F: average number of filled teeth, DMFS: decayed, missing, filled surfaces.

Figure 1 summarises the results of the dental and periodontal examinations according to the study groups.

The average plaque score was 0.62 in the control group, whereas in the infertile group, this number was 0.52, which indicated a statistically significant gap (*P* = 0.028).

The frequency of BOP % (*P* = 0.938), or the mean of PD (*P* = 0.118) did not differ significantly between the two groups.

Overall, 53 women had BOP ≥ 50% and at least one site and ≥ 4mm pocket depth in the examined group, from which 24 women were pregnant, and 29 were infertile. This did not show a statistically significant difference between the groups (*P* = 0.722). Out of this number, 48 women had BOP ≥ 50% and at least two sites with a minimum 4 mm pocket depth, numerically 21 control and 27 infertile. This was also similarly distributed (*P* = 0.944).

The number of filled teeth and DMFS showed a significant difference (fillings: *P* = 0.017, DMFS: *P* = 0.022). All the other indices were similar in the control and infertile groups, not showing a significant difference.

Table 2 compares the various parameters on the basis of the age of the examined women; the following three age categories were created: 18–25 years, 26–32 years, and 33–40 years. DMFT data were generally better in pregnant women. Statistically significant differences were found in the plaque score in the infertile group (*P* = 0.042), with the highest plaque scores found in the youngest group of women. All other parameters were divided similarly in both groups.

**Table 2:** Periodontal and dental parameters according to age groups

Age groups	Plaque score (average)		PD (average)		BOP %		DMF		DMFS		Radices	
	infertile	control	infertile	control	infertile	control	infertile	control	infertile	control	infertile	control
**18–25**	1.60	0.79	1.62	2.13	14.28	53.64	11.0	7.69	15	10.46	0	0.15
**26–32**	0.75	0.72	2.1	2.21	42.64	40.79	10.56	9.16	19.06	17.12	0.19	0.13
**33–40**	0.61	0.71	2.0	2.16	41.86	35.13	10.65	9.96	23	20.14	0.06	0.05
*P* **value**	**0.042**	0.212	0.582	0.828	0.493	0.085	0.992	0.465	0.320	0.073	0.524	0.641


Comparing the periodontal and dental parameters according to place of residence in Table 3, a significant difference (*P* = 0.046) was found in the case of BOP in the infertile group, where villagers showed worse results. Considering the dental parameters, DMFS showed a statistically significant gap (*P* = 0.039) in the control group; villagers had a larger DMFS index. All other parameters were similar.

**Table 3:** Periodontal parameters according to place of residence

Residence	Plaque score (average)		PD (average, mm)		BOP %		DMF		DMFS		Radices	
	infertile	control	infertile	control	infertile	control	infertile	control	infertile	control	infertile	control
**city**	0.66	0.76	2.06	2.14	38.76	42.53	10.3	8.9	20.6	15.3	0.08	0.08
**village**	0.72	0.82	2.16	2.32	50.1	37.11	11.5	10	23.6	23.25	0.17	0.25
*P* **value**	0.611	0.643	0.343	0.161	**0.046**	0.481	0.241	0.477	0.322	**0.039**	0.459	0.129


Analysing various factors based on educational level 3 groups were distinguished: primary/technical school, grammar school (with matriculation) and higher education (university). The following results were obtained: Statistically significant difference was found for average plaque score, both in the infertile (*P* = 0.001) and the control (*P* = 0.006) group, PD average in the infertile group (*P* = 0.070) and BOP in the infertile group (*P* = 0.004). The number of radices also showed a significant difference in the infertile group (*P* = 0.034). All parameters showed an improvement with a higher educational level (Table 4).

**Table 4:** Dental and periodontal parameters according to educational level

Educational level	Plaque score (mean)		PD (mean)		BOP %		DMF		DMFS		Radices	
	infertile	control	infertile	control	infertile	control	infertile	control	infertile	control	infertile	control
**Primary/technical school**	1.04	1.16	2.34	2.28	54.02	51.85	11.28	10.7	23.35	20.45	0.43	0.27
**Grammar school**	0.72	0.70	2.07	2.25	48.69	40.29	10	10	22.68	18.76	0.04	0.10
**Higher education (university)**	0.55	0.68	2.02	2.09	34.39	38.87	10.7	8	20.15	14.27	0.04	0.06
*P* **value**	**0.001**	**0.006**	**0.070**	0.232	**0.004**	0.286	0.639	0.205	0.584	0.227	**0.034**	0.235


Using backward elimination in logistic regression, the model shown in Table 5 was obtained. These results show that the risk of infertility increased significantly with age and with higher DMFS. The risk of infertility was 3.4 times higher for smokers compared to non-smokers (*P* = 0.076), and 3.6 times higher for those who consumed alcohol (*P* = 0.001, 95% CI (1.71–7.93)).

**Table 5:** Multiple logistic regression backward elimination model of simultaneous factors

	Sig.	Exp(B) = OR odds ratio	95% CI for EXP (B)	
			Lower	Upper
**Age**	0.004	1.115	1.035	1.202
**DMFS**	0.048	1.035	1.000	1.070
**Smoking**	0.076	**3.437**	0.881	13.411
**Alcohol consumption**	0.001	**3.687**	1.713	7.934
**Sulcus depth (mm)**	0.044	0.381	0.149	0.972
**Constant**	0.092	0.080		


### DISCUSSION

Many research can be found investigating the relationship between periodontal disease and several health conditions. The purpose of our study was to investigate any potential associations between female infertility and periodontal disease in South East Hungary. Contrary to some expectations and previous research, where researchers found that infertile women presented worse periodontal parameters than the control group,^21,41^ our results showed no discernible differences in periodontal health between pregnant and infertile women. However, in these earlier studies, none of the control group was confirmed to be pregnant at the time of the examination, raising uncertainty about their fertility status during the examination, especially considering the potential for secondary infertility.^1,4^

Our investigation found no significant differences in periodontal health indicators, such as probing depth and bleeding on probing, between the groups of pregnant and infertile women. However, according to our results, the periodontal status of the examined infertile women was similar to that of the pregnant group, which may be attributed to early pregnancy-related hormonal changes.^9,10^ These results suggest that, within the limitations of our study, periodontal disease may not be a distinguishing factor in female infertility.

On the other hand, some statistically significant differences were revealed between the two groups.

Regarding the sociodemographic data, a significant difference was discovered in the average age of the subjects in the control and infertile groups. It is a well-known fact that older age negatively affects fertility. Women’s fertility starts to decline gradually and noticeably around the age of 32, with a more rapid decline occurring after the age of 37.^2^

In this research, infertile women were older (> 3 years on average) than their pregnant counterparts, which supports the former statement.

Our study revealed another significant difference between the two groups in the case of alcohol consumption. All pregnant women reported no alcohol consumption at the time of the examination; some of them admitted occasional alcohol consumption prior to conception.

According to the World Health Organization’s latest Global Status Report on Alcohol, the consumption of alcohol is reflected in the fact that over 40% of the global population aged 15 and older consumes alcohol.^3^ Former studies found that alcohol has numerous harmful effects on general health, including acting as a teratogen, being genotoxic and a carcinogen, causing hepatotoxicity, being neurotoxic to the brain, leading to injuries, contributing to cardiovascular diseases, increasing the risk of communicable diseases such as HIV, tuberculosis, pneumonia, and COVID-19, and potentially affecting the risk of overweight and obesity.^3^

In our study, infertile women consumed alcohol significantly more frequently than pregnant women before pregnancy, which indicates that alcohol consumption may have a negative impact on fertility.

Considering the periodontal data of pregnant and infertile women, a significant difference could only be found in the case of plaque score, which can be influenced by hormonal levels. Infertile women had a lower plaque score than pregnant women. Researchers found that in the early stages of pregnancy, some mothers may have strong cravings for certain foods, especially carbohydrates. Additionally, they often experience gum bleeding, as well as nausea, which can discourage them from maintaining their oral hygiene at a high level by brushing their teeth regularly. This results in an accumulation of bacterial plaque.^40^

Other periodontal parameters such as BOP, BOP%, PD, BOP ≥ 50% and a ≥ 4 mm deep periodontal pocket of at least at one site/two sites did not show a significant difference between the control and infertile group. Moreover, no significant difference was found when examining ‘poor periodontal status’, which was considered to be positive if a 4 mm deep periodontal pocket was found at least at one site, also referred to as ‘critical probing depth’^17^ and ‘BOP frequency’, more specifically BOP at ≥ 50% of teeth,^16^ as these measures primarily indicate the current extent of inflammation. These criteria for poor periodontal status were selected because a pocket probing depth of less than 4 mm can be considered within the range of normal variation,^15^ moreover, a 4 mm or deeper pocket signifies a level of disease where the potential for attachment gain after treatment is significantly reduced compared to a 3 mm pocket depth.^16^ The other criterion, BOP, was chosen because numerous studies have demonstrated that it is a reliable indicator of periodontitis activity and inflammation.^14^

Although clinical attachment loss (CAL) is often assessed as an indicator of inflammation in many studies, we did not include CAL in our analysis. The reason for this is that CAL primarily reflects past periodontal conditions and may result from noninfectious factors, such as improper tooth brushing techniques. Furthermore, not all sites affected by gingivitis progress to develop CAL.^30^

Additionally, CAL occurs more often in the older population.^20^ The lack of significant differences related to the aforementioned factors could be attributed to the small sample size and the fact that the women in the control group were pregnant. Also, it is well established that the periodontium undergoes changes during pregnancy.^9^ The prevalence and severity of gingival disease are significantly elevated in pregnant women, with a noticeable rise starting in the second month of gestation and peaking in the eighth month.^19^ The exacerbation of gingivitis is attributed to hormonal fluctuations in oestrogen and progesterone, coupled with alterations in oral microbiota and a diminished immune response.^33^

This study faced some limitations, primarily due to the difficulty in establishing an appropriate control group. This was challenging because infertility can also be secondary, meaning some women may already have a child but face difficulties conceiving again.^1,4^ To ensure the control group comprised women who were not infertile, we required participants who were currently pregnant in the early stages of pregnancy, before the development of pregnancy gingivitis. Additionally, pregnancy gingivitis can develop in some women before the second trimester,^10^ further complicating the selection of an appropriate control group. Consequently, it could be another explanation that no connection could be discovered between periodontal disease and female infertility in this study; however, the control group can also be a strength of the study.

A former research revealed that pregnant women tend to brush their teeth less frequently than non-pregnant women, and consume more snacks during the day, which leads to the deterioration of the teeth.^11^ However, our study did not confirm these results. In our study population, only filled teeth and the DMFT index showed a significant difference, and infertile women had worse dental status, which may be connected with older age.

In a study involving 350 patients, increased Community Periodontal Index of Treatment Needs (CPITN) scores and DMFT values were significantly correlated with lower levels of education (*P* < 0.05), which was also supported in our study. Additionally, subjects with higher educational status exhibited significantly better periodontal parameters (*P* < 0.05). Therefore, the oral health status, in terms of periodontal disease and dental caries, appears to be correlated with patients’ educational level.^35^ Our study confirmed this fact, both in the infertile and in the pregnant group, a higher educational level indicated better results in dental parameters such as DMFS, and periodontal parameters, namely BOP%. Another possible limitation of the study is that individuals with a very low socioeconomic status often do not attend infertility investigations, despite the fact that these services are provided free of charge. As a consequence, it is likely that individuals with extremely low income who live under very poor conditions were underrepresented or not included in our study sample, affecting the results.

Our research topic could be further investigated with a prospective study design, in which the dental and periodontal status of women desiring a child, who are otherwise healthy and have a fertile partner, is assessed at baseline and subsequently followed for 1 year. Success of conception could then be recorded, and the groups (those who conceive and those who do not) compared accordingly.

### CONCLUSION

There have been only a few publications available which investigate the relation of periodontal disease and female infertility. In conclusion to other researchers’ previous findings, periodontal inflammation acts as a focal infection and may cause impairment in the female reproductive system. However, within the limitations of the present study, this hypothesis was not supported by the results of our research. To explore the connection between periodontal disease and female idiopathic infertility, further clinical, prospective studies with more cases are clearly needed. At the same time, we have found significant differences in other dental parameters and some sociodemographic data, and our findings further confirm the detrimental effects of advancing age, alcohol consumption, and smoking on conception.

It is a remarkable result that nearly half of the examined pregnant women presented elevated BOP values as a sign of gingivitis. If untreated, periodontal status may worsen during pregnancy, which may be associated with preterm birth; therefore, early detection and management are essential.

#### Acknowledgements

##### Data availability statement

Data supporting the findings of this study are available from the corresponding author upon reasonable inquiry.

##### Funding statement

University of Szeged Open Access Fund, Grant ID:8017.

##### Ethics approval consent

This study was approved by the Regional and Institutional Human Medical Biological Research Ethics Committee in Szeged (protocol number: 16/2018-SZTE).

##### Patient consent

Each participant in the study gave their written consent prior to the examination.
